# Sexually Transmitted and Other Genital Infections in Women With Cervical Human Papillomavirus Infection

**DOI:** 10.1155/S1064744995000342

**Published:** 1995

**Authors:** B. Sikström, D. Hellberg, S. Nilsson, I. Kallings, P.-A. Mårdh

**Affiliations:** 1 Department of Obstetrics and Gynecology Mälar Hospital Eskilstuna Sweden; 2 WHO Collaborative Centre for STDs and Their Complications Uppsala University Uppsala Sweden; 3 Department of Obstetrics and Gynecology Uppsala University Uppsala S-791 82 Sweden; 4 Swedish Infectious Disease Control Stockholm Sweden; 5 Department of Obstetics and Gynaecology Falun Hospital Falun Sweden

## Abstract

*Objective:* We investigated possible correlations between latent cervical human papillomavirus infection (CHPI) and other sexually transmitted diseases (STDs).

*Methods:* Of 972 randomly selected women attending 2 family planning clinics and a youth clinic who had agreed to participate in a study concerning STDs, 66 (6.8%) had latent CHPI.

*Results:* An association was found between latent CHPI on one hand and a history of genital chlamydial infection, gonorrhea, recurrent vaginal candidiasis, cervicitis, or pelvic inflammatory disease (PID) on the other, while no correlation between latent CHPI and coexistent STDs was found. No correlation of latent CHPI to either current or past genital warts was noted. In multifactorial analyses, which included the lifetime number of sexual partners and age at first intercourse, we found that all significant associations except a history of gonorrhea vanished.

*Conclusions:* In this study population, screening for other current STDs in women with latent CHPI would be of limited value.


Infectious Diseases in Obstetrics and Gynecology 3:67–72 (1995)
© 1995 Wiley-Liss, Inc.

### Sexually Transmitted and Other Genital Infections in Women With Cervical Human Papillomavirus Infection

**B. Sikström, D. Hellberg, S. Nilsson, I. Kallings, and P.-A. Mårdh**

*Department of Obstetrics and Gynecology, Mälar Hospital, Eskilstuna (B.S.), WHO Collaborative Centre for STDs and Their Complications (P.-A.M.), and Department of Obstetrics and Gynecology (B.S.), Uppsala University, Uppsala, Swedish Infectious Disease Control (I.K.), Stockholm, and Department of Obstetics and Gynaecology (D.H., S.N.), Falun Hospital, Falun, Sweden*

ABSTRACT

***Objective:*** **We investigated possible correlations between latent cervical human papillomavirus infection (CHPI) and other sexually transmitted diseases (STDs).**

***Methods:*** **Of 972 randomly selected women attending 2 family planning clinics and a youth clinic who had agreed to participate in a study concerning STDs, 66 (6.8%) had latent CHPI.**

***Results:*** **An association was found between latent CHPI on one hand and a history of genital chlamydial infection, gonorrhea, recurrent vaginal candidiasis, cervicitis, or pelvic inflammatory disease (PID) on the other, while no correlation between latent CHPI and coexistent STDs was found. No correlation of latent CHPI to either current or past genital warts was noted. In multifactorial analyses, which included the lifetime number of sexual partners and age at first intercourse, we found that all significant associations except a history of gonorrhea vanished.**

***Conclusions:*** **In this study population, screening for other current STDs in women with latent CHPI would be of limited value.** © 1995 Wiley-Liss, Inc.

KEY WORDS

Genital HPV infection, gential warts, genital chlamydial infection, gonorrhea

Genital warts are mainly caused by infection with human papillomavirus (HPV) types 6 and 11, while cervical human papillomavirus infection (CHPI) is mainly caused by HPV types 16, 18, 31, 33, and 35.

It has been speculated that the presence of a coexistent infection of the genital epithelium increases the susceptibility to HPV or that a coexistent sexually transmitted disease (STD) can activate a latent CHPI.[1] Indirect evidence of a correlation between latent CHPI and STDs is the high prevalence of latent CHPI in STD clinic patients[2–4] compared with the general population, with an estimated prevalence of 6–12%.[5,6] Although a history of STD is common in latent CHPI patients,[7] less well established is whether a concurrent STD is more frequent in latent CHPI patients.

The purpose of our study was to examine any correlations between latent CHPI and the presence or a history of STDs, i.e., *Chlamydia trachomatis, Neisseria gonorrhoeae,* genital herpes, or genital warts. Furthermore, we sought correlations of current latent CHPI and previously diagnosed cervicitis, pelvic inflammatory disease (PID), or recurrent genital candidiasis.

### MATERIALS AND METHODS

Between November 1989 and January 1991, 3–4 women per day were enrolled in the study at the family planning clinics at Eskilstuna and Danderyd

**Address correspondence/reprint requests to Dr. Dan Hellberg, Department of Obstetrics and Gynaecology, Falun Hospital, S-791 82 Falun, Sweden.**

**Dr. B. Sikström is now at the Carolina Clinic, Uppsala, Sweden.**

Received October 7, 1994

Accepted May 19, 1995

Hospitals (Stockholm) and at the youth clinic in Eskilstuna. Eskilstuna is a town of 90,000 inhabitants located 110 km southwest of the capital city, Stockholm (1.5 million inhabitants).

A selected number of midwives were trained for the particular tasks involved in the study. Women with predetermined positions on the outpatient list were asked by midwives if they would like to participate in the study. The midwives were not involved in the patient scheduling, so the identities of the participants were unknown to the midwives. The participants were added to the outpatient list consecutively when they booked a time so as to avoid selection bias.

The midwives conducted the structured personal interviews and collected the laboratory samples. The information on previous STDs and genital infections was checked in the original patient records to avoid memory bias.

Of the 1,077 women who were asked, 1,011 (93.9%) agreed to participate. This high participation rate likely reflects a great interest in the study design, which offered the detection of STDs and other gynecologic infections. HPV screening was performed in all women but the first 39 (3.9%) women who were enrolled at the beginning of the study before the test reagents became available. Thus, 972 women were available for analysis. The 906 women with negative HPV tests made up a comparison group (COMP).

Occasionally, there were some missing values that rarely exceeded 1% of the study population. These are not included in the tables.

No economic compensation was offered to the participating women. The anonymity of the participants was guaranteed to ensure as honest answers as possible. No identifying details were included in either the patient record forms or computerized data. Only the authors had access to the patient codes.

In all participants, urethral and endocervical specimens for the isolation of *C. trachomatis* were collected with nontoxic, cotton-tipped swabs and transported in a sucrose phosphate buffer (2-SP) with 10% fetal calf serum. The cultures were grown on cycloheximide-treated McCoy cells.[8] Direct immunofluorescence using labeled monoclonals (Micro Track, SYVA, San Jose, CA) were also used in all women for the detection of *C. trachomatis* inclusions. The cervical samples for enzyme immunoassay were collected by swabs provided by the kit producer (Chlamydiazyme, Abbott, or Chlamydia EIA, SYVA). Positive Chlamydiazyme tests were confirmed by Abbott's Chlamydia Confirmatory Test.[9] A positive test with either method was regarded as proof of a genital chlamydial infection. Urethral and endocervical specimens were collected for the culture of *N. gonorrhoeae.*[10] For the detection of *Trichomonas vaginalis,* wet smear microscopy was performed. The cultures for yeast fungi were made on Rogosa agar from swab samples from the posterior vaginal fornix and analyzed by serum tests for the distinction of *Candida albicans*. Serological screening tests for human immunodeficiency virus (HIV) were made by two enzyme-linked immunosorbent assay (ELISA) methods: Wellcozyme HIV-1 recombinant EIA (Wellcome Diagnostics, Dartford, England) and Enzygnost anti HIV-1,2 EIA (Behringwerke AG, Marburg, Germany).

For the detection and typing of HPV DNA, a cell sample was collected from the ectocervix by means of a brush device (Cytobrush, Medscand, Malmö, Sweden) and washed with 1ml of aqueous lytic buffer (Oncor®, Inc., Gaithersburg, MD) in a 2-ml microfuge tube.[11] The tubes were mailed to the laboratory at ambient temperature and stored at 4°C until analyzed. The HPV typing was made by Southern blot. Sodium dodecyl sulfate was added to the specimens, after which they were digested with proteinase K and incubated at 50°C for 15 min. Residual peptides were salted out by adding a "protein precipitating agent" (Oncor®) retaining the supernatant. After vortexing and centrifugation, the DNA was precipitated from this supernatant with 3 volumes of ethanol at room temperature. After careful washings twice with ethanol, the preparations with resultant DNA pellets were dissolved overnight at 52°C using Tris-EDTA buffer. After digestion with 2 restriction enzymes (Bam H1 and Pst 1) and electrophoresis in 1% agarose gel, the separated DNA fragments were first depyrinated with hydrochloric acid and then denatured with sodium hydroxide. The blotting onto a nylon membrane was accomplished with limited vacuum using the Probe Tech electrophoresis and transfer equipment (Oncor®). The hybridization was then performed using a mixture of subgenomic probes selected to label only one band of a defined size for each of the HPV types studied (Oncor Human Papillomavirus Typing System®). The cases with

**TABLE 1. Distribution of age among women with latent CHPI and a comparison group (COMP)***

| Age (years) | Latent CHPI (%) (N = 66) | COMP (%) (N = 905) |
|---|---|---|
| 15–20 | 15 (22.7) | 151 (16.7) |
| 20–24 | 26 (39.4) | 331 (36.6) |
| 25–29 | 12 (18.2) | 201 (22.2) |
| 30–34 | 9 (13.6) | 104 (11.5) |
| 35–39 | 1 (1.5) | 53 (5.9) |
| 40–48 | 3 (4.6) | 65 (7.2) |
| Total | 66 (100.0) | 905 (100.1) |

*$P = 0.35$.

distinct bands, however unrelated to any of the standards, were designated as unknown types.

The samples were obtained in the following order: general culture, gonorrhea culture, chlamydia culture, Chlamydiazyme, and sample for HPV typing.

The results were computerized and analyzed with the JMP statistical program.[12] The initial significance tests were performed by chi-square for nominal variables (Pearson and likelihood ratio) and t-test for continuous variables. In order to adjust for age differences and to assess the simultaneous effect of more than one variable, we used multiway frequency tables analyzed by means of logistic regression (analysis of log likelihood) to identify and check for possible confounding.

### RESULTS

Sixty-six (6.8%) of the 972 women were positive for one or more of the following HPV types: 4 HPV6, 4 HPV 11, 25 HPV 16, 10 HPV 18, 14 HPV31, 5 HPV33, 5 HPV 35, and 8 unknown HPV types. Nine women had mixed HPV infections.

The mean age for the women with latent CHPI was 24.5 years (SD 0.87), while it was 26.0 years (SD 0.23) for the COMP group ($P = 0.10$). The age range was 15–48 years. The age distribution is given in Table 1.

All women were sexually active, but partner changes were significantly more frequent in ages 15–19 years ($P = 0$), with 36% reporting 2 or more sexual partners during the previous 6 months. This frequency continuously decreased with every 5-year group to 7% of the women above 35 years of age. The frequency of having had sex with a casual partner during the previous month was significantly different only among women in ages 30–34 years (1.6%, $P = 0.02$). In the other age groups, this figure varied from 5.4% (above 35 years) to 9.3% (25–29 years) (not shown in Table 1).

There were no HIV-positive women in the study. Only one case of trichomoniasis (0.1%) was noted.

For genital chlamydial infection, gonorrhea, genital warts, genital herpes, and vaginal candidiasis, the following 3 variables were detailed: history, current infection, and the occurrence of more than one episode of an infection (Table 2).

There were 83 (8.8%) chlamydia-positive women. No significant difference was noted in the prevalence between the latent CHPI and COMP groups. A history of chlamydial infection was significantly more frequent in the latent CHPI (30.3%) than in the COMP (15.8%) group, but no difference was noted between the 2 groups in a history of more than one episode of chlamydial infection (including the current episode).

Four percent of the study population reported having previously had gonorrhea. Such a history was noted significantly more often in women with latent CHPI than in women in the COMP group ($P = 0.006$).

There were 39 cases of current genital warts in the vagina, on the vulva, or in the perianal area, equally distributed in the 2 groups of women, as was also the case for a history of genital warts. In 95% of the cases, the diagnosis had been made by a physician. Of the 8 cases who were current carriers of HPV types 6 and 11 in the cervix, 2 had current genital warts, which was a significantly increased frequency ($P = 0.003$) compared with the women in the COMP group, and 1 had a history of genital warts.

No difference between the latent CHPI and COMP groups was noted with regard to genital herpes.

A history of genital candidiasis was recorded in 372 women (38.3%), with no difference between the latent CHPI and COMP groups. Of those who had such a history, the mean number of episodes was significantly higher in the latent CHPI than in the COMP group. *C. albicans* was isolated from 71 (7.9%) of the 972 women (yeast cultures were not performed in 67 women), with no significantly different prevalence in the 2 groups of women studied.

TABLE 2. Occurrence of STDs and vulvovaginal candidiasis among women with latent CHPI and a comparison group (COMP)

| | Latent CHPI (%) (N = 66) | COMP (%) (N = 906) | P |
|---|---|---|---|
| History of chlamydial infection | 20 (30.3) | 143 (15.8) | 0.002 |
| Current chlamydial infection | 4 (6.1) | 79 (9.0) | 0.40 |
| >1 episode of chlamydial infection | 2 (3.0) | 31 (3.4) | 0.86 |
| History of gonorrhea | 7 (10.6) | 33 (3.6) | 0.006 |
| Current gonorrhea | 0 | 1 (0.1) | 0.75 |
| >1 episode of gonorrhea | 1 (1.5) | 3 (0.3) | 0.15 |
| History of genital warts | 4 (6.1) | 69 (7.6) | 0.63 |
| >1 episode of genital warts | 1 (1.5) | 9 (1.0) | 0.42 |
| Signs of genital warts | 3 (4.6) | 36 (4.0) | 0.82 |
| History of herpes | 3 (4.6) | 32 (3.5) | 0.68 |
| Mean no. of episodes of herpes | 2.7 | 11.0 | 0.50 |
| Signs of herpes | 0 | 3 (0.3) | 0.81 |
| History of candidiasis | 27 (40.9) | 345 (38.1) | 0.65 |
| Mean no. of episodes of candidiasis | 7.8 | 3.6 | 0.01 |
| Signs of candidiasis | 7 (12.1) | 64 (7.6) | 0.22 |

TABLE 3. History of cervicitis and any STD among women with latent CHPI and a comparison group (COMP)

| | Latent CHPI (%) (N = 66) | COMP (%) (N = 906) | P |
|---|---|---|---|
| History of PID | 9 (13.6) | 55 (6.1) | 0.02 |
| Medical treatment for PID | 9 (100.0) | 54 (98.2) | 0.58 |
| History of cervicitis | 11 (16.7) | 69 (7.6) | 0.01 |
| Medical treatment for cervicitis | 10 (90.9) | 55 (80.9) | 0.58 |
| History of any STD | 28 (42.4) | 228 (25.2) | 0.003 |

Cervical cytology (not shown in Table 2) with changes suggestive of HPV infection or cervical intraepithelial neoplasia was positive in 18 women, of whom 9 were HPV negative. The inclusion of these 9 women in the latent CHPI group made no difference in the significance tests. However, the frequency of current genital warts increased to 6.7% and the frequency of current genital chlamydial infection increased to 8.0% in the women in the latent CHPI group. The corresponding values for the COMP group were 3.8% and 8.7%, respectively.

There was a significant difference with regard to a history of any STD, i.e., genital chlamydial infection, gonorrhea, genital warts, trichomoniasis, or genital herpes, which was reported by 42% of those in the latent CHPI group and 25% in the COMP group (Table 3).

A history of cervicitis and PID was more than twice as frequent among those with latent CHPI than among those in the COMP group. In 17 (26.6%) of the 64 cases with a history of PID, the diagnosis had been laparoscopically verified. There was no difference between the 2 groups. In the remaining cases, only clinical criteria had been used to establish the diagnosis. Thirty-nine (59.1%) of the patients with a PID history had received hospital care.

In the logistic regression analyses with adjustment for age, age at first intercourse, and lifetime number of sexual partners, we found a significant correlation between latent CHPI and a history of gonorrhoea, but not for chlamydial infection, which vanished ($P = 0.03$ and 0.12, respectively), as did a history of any STD ($P = 0.12$) and of recurrent candidiasis ($P = 0.06$). A history of both PID and cervicitis lost its significance ($P = 0.09$ and 0.10, respectively) when a history of any STD was included in the analyses.

### DISCUSSION

A principal finding of our study was the lack of a correlation between latent CHPI and coexistent STDs, e.g., genital clamydial infection and genital warts. Thus, in the population we studied, there would have been no benefit in selecting women with latent CHPI as a prescreening method for other STDs or vice versa. Our results did not support the theory of the ability of other STDs to activate a latent CHPI or to make the genital mucosa more susceptible for the acquisition of HPV.

An unexpected finding was the lack of a significant difference for the prevalence of latent CHPI between ages, although the mean age for women with latent CHPI was 1.5 years less than for women in the COMP group. One reason for the relatively low age difference between the 2 groups studied might be the comparably high sexual activity present in the older age groups.

The possibility that "STD core members" function as a reservoir has been used to support the prospect of a relationship between latent CHPI and other STDs.[13] The "core members" have been defined as a group of males and females who harbor specified STDs and, after treatment, often reinfect each other. As a result, the frequency of the STDs will not decrease in this population. Our study was not able to define a single core group for the current STDs that were studied.

The strong correlation in our study between a current latent CHPI and a history of genital chlamydial infection or gonorrhea has also been found in other studies,[14,16] as well as in 2 previous studies[15,17] not reporting a correlation between latent CHPI and coexistent chlamydial cervicitis. Yliskoski and coworkers[18] found that a concomitant cervicitis had no influence on the clinical course of latent CHPI.

Both latent CHPI and genital chlamydial infection involve the squamous epithelium in the transitional zone. It has been thought that ectopy facilitates the establishment of both infections. It remains to be proved, however, that the same is true for chlamydial infections.[19] In our study population, it was not true for latent CHPI (unpublished results).

In our multifactorial analyses, only a history of gonorrhea remained significant after the adjustment for age, sexual behavior, and a history of any STD. All other associations were only covariables for sexual promiscuity, an expected result confirming that the STDs studied here have the same mode of transmission. We have no explanation for why a history of gonorrhea remained significant after adjustment. There might have been behavioral factors that we could not control for.

One current STD is a well-known risk factor for other STDs.[13] An unexpected finding was the lack of a significant association between latent CHPI and current genital warts or between latent CHPI and a history of genital warts. The diagnosis of genital warts, often suspected by the woman herself, is usually clinically uncomplicated, although suspected warts might represent other conditions, mostly benign tumors. In this study, the disgnoses were made by gynecologists, venereologists, or by physicians of other specialities in 95% of the cases.

Our study confirmed an association of latent CHPI with HPV types 6 and 11 and genital warts. Moscicki and coworkers[15] also found a relationship between latent CHPI and genital warts. In their study, 24% of the cases of latent CHPI were caused by HPV types 6 and 11 compared with 12% in our study. In those patients who were positive for HPV types 6 and 11, genital warts were found in 58%, whereas in those patients with other HPV types, visible warts were noted in only 22%. Law and coworkers[20] found no correlation between latent CHPI and coexistent genital warts, which is in contrast to still another study[21] reporting an association between a history of genital warts and latent CHPI. These contradicting results raise the question of whether latent CHPI and genital warts, despite being caused by the same virus, are not more closely linked to each other than to other STDs from an epidemiological and behavioral point of view.

Although most studies on latent CHPI and genital warts have clearly demonstrated the dominance of a sexually transmitted route,[22,23] other transmission routes have been proposed, for instance, by fomites. In a recent study, the habit of bathing in hot tubs was strongly correlated after adjustment for age and sexual risk behavior to the occurrence of genital warts.[24]

Both a history of PID and a history of cervicitis were associated with latent CHPI, but the significance vanished after the adjustment for sexual variables and a history of any STD. Although this finding indicates that sexual risk behavior is the common denominator for these conditions, no conclusions can be drawn as to whether cervicitis or PID predisposes a patient to latent CHPI or vice versa. The clinical diagnosis of PID is difficult to establish with any degree of accuracy as, e.g., cervicitis can mimic PID. However, both diseases were noted to be markers for a subsequent risk of acquiring latent CHPI.

Our study showed that, in the population reported, a previous STD was a risk factor for current latent CHPI, but provided no evidence of an increased prevalence of coexistent STDs in women with latent CHPI.

### ACKNOWLEDGMENT

This study was supported by a grant from Arbetsmarknadens Försäkringsaktiebolag.

### REFERENCES

1. von Krogh G: Genitoanal papillomavirus infection: Diagnostic and therapeutic objectives in the light of current epidemiological observations. Int J STD AIDS 2:391–404, 1991.
2. Byrne MA, Taylor-Robinson D, Anderson MAC, Mason P, Harris JR: Value of colposcopy in sexually transmitted diseases clinic based on first year's experience. Genitourin Med 65:42–45, 1989.
3. McKinnon KJ, Ford RM, Hunter JC: High prevalence of human papillomavirus and cervical intraepithelial neoplasia in a young Australian STD population. Int J STD AIDS 2:276–279, 1991.
4. Csango PA, Skuland J, Nilsen A, Stray-Pedersen B, Jagars G: Papillomavirus infection among abortion applicants and patients at a sexually transmitted disease clinic. Sex Transm Dis 19:149–153, 1992.
5. Beutner KR, Becker TM, Stone KM: Epidemiology of human papillomavirus infections. Dermatol Clin 9:211–218, 1991.
6. Kjaer SK, Jensen OM: Comparison studies of HPV detection in areas at different risk for cervical cancer. In Munoz N, Bosch FX, Shah KV, Meheus A (eds): The Epidemiology of Cervical Cancer and Human Papillomavirus. Lyon: IARC, pp 243–249, 1992.
7. Moscicki A-B: Human papillomavirus infections. Adv Pediatr 39:257–281, 1992.
8. Ripa KT, Mårdh P-A: Cultivation of *Chlamydia trachomatis* in cyclohexamide-treated McCoy cells. J Clin Microbiol 6:328–331, 1977.
9. Mårdh P-A, Elbagir AN, Stenberg K: Use of blocking reagent to confrim enzyme immunoassay results in samples from patients with chlamydial conjunctivitis. Eur J Clin Microbiol Inf Dis 9:292–293, 1990.
10. Mårdh P-A, Mårtensson D, Soltesc LV: An effective, simplified medium for the culture of *Neisseria gonorrhoeae*. Sex Transm Dis 5:10–13, 1978.
11. Hjerpe A, Lindh E, Bistoletti P, George J, Groff D: Use of cervical Cytobrush samples for dot-blot detection and Southern blot typing of human papillomaviruses using subgenomic probes. Anal Quant Cytol Histol 12:299–305, 1990.
12. SAS Institute, Inc.: JMP User's Guide. Cary, NC: SAS Institute, Inc., 1989.
13. Padian N, Hitchcock PJ, Fullilove RE, Kohlstadt V, Brunham R: Report of the NIAD study group on integrated behavioral research for prevention and control of sexually transmitted diseases. Sex Transm Dis 17:200–204, 1990.
14. Daling JR, Weiss NS, Sherman KJ: History of genital warts in a selected population. Lancet 1:157–158, 1984.
15. Moscicki A-B, Palefsky J, Gonzales J, Schoolnik GK: Human papillomavirus infection in sexually active adolescent females: Prevalence and risk factors. Pediatr Res 28:507–513, 1990.
16. Andersson-Ellström A, Forssman L: Genital papillomavirus infection in women treated for chlamydial infection. Int J STD AIDS 3:42–45, 1992.
17. Syrjänen K, Mäntyjärvi R, Väyrynen M, Castren O, Yliskoski M, Saarikoski S: Chlamydial cervicitis in women followed up for human papillomavirus (HPV) lesions of the uterine cervix. Acta Obstet Gynaecol Scand 64:467–471, 1985.
18. Yliskoski M, Tervahauta A, Saarikoski S, Mäntyjärvi R, Syrjänen K: Clinical course of cervical human papillomavirus lesions in relation to coexistent cervical infections. Sex Transm Dis 19:137–139, 1992.
19. Rahm VA, Odlind V, Pettersson R: *Chlamydia trachomatis* in sexually active teenage girls. Factors related to genital chlamydial infection: A prospective study. Genitourin Med 67:317–321, 1991.
20. Law CL, Merianos A, Grace J, Rose BR, Thompson CH, Cossart: Clinical and virological associations between external anogenital warts and cervical HPV infection in an STD population. Int J STD AIDS 2:30–36, 1991.
21. Syrjänen K, Väyrönen M, Castren O, et al.: Sexual behaviour of women with human papillomavirus (HPV) lesions of the uterine cervix. Br J Vener Dis 60:243–248, 1984.
22. Schneider A, Koutsky LA: Natural history and epidemiological features of genital HPV infection. In Munoz N, Bosch FX, Shah KV, Meheus A (eds): The Epidemiology of Cervical Cancer and Human Papillomavirus. Lyon: IARC, pp 25–52, 1992.
23. Daling JR, Scherman KJ, Weiss NS: Risk factors for condyloma acuminatum in women. Sex Transm Dis 13: 16–18, 1986.
24. Hellberg D, Borendal N, Sikström B, Nilsson S, Mårdh P-A: Comparison of women with cervical human papillomavirus infection and genital warts. Genitourin Med 71: 88–91, 1995.

## References

[PDF_00517] Andersson-Ellström A., Forssman L. (1992). Genital papillomavirus infection in women treated for chlamydial infection.. Int J STD AIDS.

[PDF_00479] Beutner K. R., Becker T. M., Stone K. M. (1991). Epidemiology of human papillomavirus infections.. Dermatol Clin.

[PDF_00467] Byrne M. A., Taylor-Robinson D., Anderson M. A., Mason P., Harris J. R. (1989). Value of colposcopy in sexually transmitted diseases clinic based on first year's experience.. Genitourin Med.

[PDF_00475] Csángó P. A., Skuland J., Nilsen A., Pedersen B. S., Jagars G. (1992). Papillomavirus infection among abortion applicants and patients at a sexually transmitted disease clinic.. Sex Transm Dis.

[PDF_00547] Daling J. R., Sherman K. J., Weiss N. S. (1986). Risk factors for condyloma acuminatum in women.. Sex Transm Dis.

[PDF_00511] Daling J. R., Weiss N. S., Sherman K. J. (1984). History of genital warts in a selected population.. Lancet.

[PDF_00550] Hellberg D., Borendal N., Sikström B., Nilsson S., Mårdh P. A. (1995). Comparison of women with cervical human papillomavirus infection and genital warts. I. Some behavioural factors and clinical findings.. Genitourin Med.

[PDF_00499] Hjerpe A., Lindh E., Bistoletti P., George J., Groff D. (1990). Use of cervical Cytobrush samples for dot-blot detection and Southern blot typing of human papillomaviruses using subgenomic probes.. Anal Quant Cytol Histol.

[PDF_00533] Law C. L., Merianos A., Grace J., Rose B. R., Thompson C. H., Cossart Y. E. (1991). Clinical and virological associations between external anogenital warts and cervical HPV infection in an STD clinic population.. Int J STD AIDS.

[PDF_00471] McKinnon K. J., Ford R. M., Hunter J. C. (1991). High prevalence of human papillomavirus and cervical intraepithelial neoplasia in a young Australian STD population.. Int J STD AIDS.

[PDF_00487] Moscicki A. B. (1992). Human papillomavirus infections.. Adv Pediatr.

[PDF_00513] Moscicki A. B., Palefsky J., Gonzales J., Schoolnik G. K. (1990). Human papillomavirus infection in sexually active adolescent females: prevalence and risk factors.. Pediatr Res.

[PDF_00492] Mårdh P. A., Elbagir A. N., Stenberg K. (1990). Use of blocking reagent to confirm enzyme immunoassay results in chlamydial conjunctivitis.. Eur J Clin Microbiol Infect Dis.

[PDF_00496] Mårdh P. A., Mårtensson D., Soltesz L. V. (1978). An effective, simplified medium for the culture of Neisseria gonorrhoeae.. Sex Transm Dis.

[PDF_00506] Padian N., Hitchcock P. J., Fullilove R. E., Kohlstadt V., Brunham R. (1990). Report of the NIAID Study Group on Integrated Behavioral Research for Prevention and Control of Sexually Transmitted Diseases. Part I: Issues in defining behavioral risk factors and their distribution.. Sex Transm Dis.

[PDF_00529] Rahm V. A., Odlind V., Pettersson R. (1991). Chlamydia trachomatis in sexually active teenage girls. Factors related to genital chlamydial infection: a prospective study.. Genitourin Med.

[PDF_00489] Ripa K. T., Mårdh P. A. (1977). Cultivation of Chlamydia trachomatis in cycloheximide-treated mccoy cells.. J Clin Microbiol.

[PDF_00521] Syrjänen K., Mäntyjärvi R., Väyrynen M., Castrén O., Yliskoski M., Saarikoski S. (1985). Chlamydial cervicitis in women followed-up for human papillomavirus (HPV) lesions of the uterine cervix.. Acta Obstet Gynecol Scand.

[PDF_00538] Syrjänen K., Väyrynen M., Castrén O., Yliskoski M., Mäntyjärvi R., Pyrhönen S., Saarikoski S. (1984). Sexual behaviour of women with human papillomavirus (HPV) lesions of the uterine cervix.. Br J Vener Dis.

[PDF_00525] Yliskoski M., Tervahauta A., Saarikoski S., Mäntyjärvi R., Syrjänen K. (1992). Clinical course of cervical human papillomavirus lesions in relation to coexistent cervical infections.. Sex Transm Dis.

[PDF_00463] von Krogh G. (1991). Genitoanal papillomavirus infection: diagnostic and therapeutic objectives in the light of current epidemiological observations.. Int J STD AIDS.

